# DNA-hydrolysing activity of IgG antibodies from the sera of patients with schizophrenia

**DOI:** 10.1098/rsob.150064

**Published:** 2015-09-16

**Authors:** Evgeny A. Ermakov, Ludmila P. Smirnova, Taisiya A. Parkhomenko, Pavel S. Dmitrenok, Nina M. Krotenko, Nikolai S. Fattakhov, Nikolay A. Bokhan, Arkadiy V. Semke, Svetlana A. Ivanova, Valentina N. Buneva, Georgy A. Nevinsky

**Affiliations:** 1 Institute of Chemical Biology and Fundamental Medicine, Siberian Division of Russian Academy of Sciences, 8 Lavrentiev Avenue, Novosibirsk 630090, Russia; 2 Novosibirsk State University, 2 Pirogova Street, Novosibirsk 630090, Russia; 3 Mental Health Research Institute, Russian Academy of Medical Sciences, 4 Aleutskaya Avenue, Tomsk 634014, Russia; 4 Pacific Institute of Bioorganic Chemistry, Far East Division, Russian Academy of Sciences, Vladivostok 690022, Russia

**Keywords:** schizophrenia, abzymes, DNA hydrolysis, autoimmune reactions

## Abstract

It is believed that damage to the membranes of brain cells of schizophrenia (SCZ) patients induces the formation of autoantigens and autoantibodies. Nevertheless, the importance of immunological changes leading to the loss of tolerance to self-antigens in the genesis of SCZ has not been established. The MALDI mass spectra of the IgG light chains of 20 healthy donors were relatively homogeneous and characterized by one peak with only one maximum. In contrast to the healthy donors, the MALDI mass spectra of IgG light chains corresponding to 20 SCZ patients demonstrated, similarly to 20 autoimmune systemic lupus erythematosus (SLE) patients, two maxima of a comparable intensity. In addition, the MALDI spectra of the IgG light chains of five SLE and four SCZ patients contained a small additional brightly pronounced peak with remarkably lower molecular mass compared with the main one. DNase autoantibodies (abzymes) can be found in the blood of patients with several autoimmune diseases, while the blood of healthy donors or patients with diseases without a significant disturbance of the immune status does not contain DNase abzymes. Here, we present the first analysis of anti-DNA antibodies and DNase abzymes in the sera of SCZ patients. Several strict criteria have been applied to show that the DNase activity is an intrinsic property of IgGs from the sera of SCZ patients. The sera of approximately 30% of SCZ patients displayed a higher content of antibodies (compared with 37% of SLE) interacting with single- and double-stranded DNA compared with healthy donors. Antibodies with DNase activity were revealed in 80% of the patients. These data indicate that some SCZ patients may show signs of typical autoimmune processes to a certain extent.

## 1. Introduction

Schizophrenia (SCZ) remains one of the most relevant problems of psychiatry. The prevalence of SCZ is approximately 1%, and this disease is the most severe mental illness inherent to the human population [1]. SCZ is a progressive mental illness occurring with polymorphic symptoms, and leading to a persistent violation of social adaptation and ability to work. In SCZ there is a violation of synaptic transmission, leading to neuronal damage and severe dysfunction [2–4]. These changes often can begin to develop *in utero* or in early childhood [5,6].

So far, there is no unified view on the aetiopathogenesis of SCZ, but there are many different theories. One widely known aspect is dysfunction of the glutamatergic system in SCZ [7–10]. It is possible that disbalance of dopamine-glutamate homeostasis in SCZ leads to the development of generalized oxidative stress in patients [11,12]. In addition, the fact of enzymatic system dysfunction involved in the metabolism of biogenic amines (indolamine, catecholamines) during mental disorders is known [13,14]. Detection of a neurotropic effect associated with damage of cell membranes was postulated [15,16]. It is believed that the damage of the cell membranes of the brain causes the formation of autoantigens and, as a consequence, autoantibodies (auto-Abs) [17–19]. Nevertheless, the importance of immunological changes leading to the loss of tolerance to self-antigens in the genesis of SCZ has not yet been established. Summarizing all existing hypotheses, one can say that the basis of SCZ may be some disturbances in the functioning of neurotransmitter systems associated with changes in the rate of synthesis or breakdown of the neurotransmitter and possible modifications of the structure of the relevant receptors. In the case of SCZ, a dysregulation between the nervous and the immune systems was observed, which can lead to changes in brain structure [20].

Despite the fact that SCZ is not attributed to classical autoimmune diseases (AIDs), immune system and immune cell dysregulation (including autoimmune processes in SCZ) are not excluded [21,22]. Therefore, the search for possible mechanisms of SCZ development is realistic.

Among all known pathologies, only systemic lupus erythematosus (SLE) is usually considered to be related to the autoimmunization of patients with DNA; the sera of SLE patients usually contain high concentrations of DNA. However, the serum of patients with several different diseases has been shown to contain DNA and anti-DNA Abs (anti-DNA antibody idiotype termed 16/6 specific for patients with SLE) [23], as well as RNA and anti-RNA Abs [24–27]. Even in the sera of healthy mammals anti-DNA Abs are detectable, but their titres vary significantly [23]. Many SLE anti-DNA Abs are directed against histone–DNA nucleosomal complexes appearing from internucleosomal cleavage during apoptosis [28].

Abzymes against transition chemical states of different reactions were studied extensively (reviewed in [29–31]). During the past two decades, it has become clear that auto-Abs from the sera of patients with different AIDs can possess enzymatic activities and that their occurrence is a distinctive feature of AIDs (reviewed in [31–34]). Similar to artificial abzymes against analogues of chemical reactions transition states [29–31], naturally occurring abzymes may be Abs raised directly against enzyme substrates acting as haptens and mimicking transition states of catalytic reactions [31–34]. On the other hand, anti-idiotypic Abs can be induced in AIDs by a primary antigen and may show some of their features, including catalytic activity [31–36].

Polyclonal natural IgG and/or IgA and IgM abzymes hydrolysing DNA, RNA, polysaccharides, nucleotides, oligopeptides and proteins from the sera of patients with several autoimmune and viral diseases were revealed (reviewed in [31–34]). Some healthy patients demonstrated abzymes with low proteolytic and polysaccharide-hydrolysing activities [31–34]. However, healthy humans and patients with many diseases causing insignificant autoimmune reactions usually lack abzymes or develop abzymes with very low catalytic activities, often at the limit of the sensitivity of detection methods [31–34]. At the same time, germline Abs from healthy humans can exhibit a high level of promiscuous, amyloid-directed and superantigen-directed activities and/or autoantigen-directed and microbe-directed specificities [37,38]. DNase abzymes from SLE [39], multiple sclerosis (MS) [40] and Bence-Jones proteins from multiple myeloma patients [41] are cytotoxic, cause nuclear DNA fragmentation and induce cell death by apoptosis.

MS is a chronic demyelinating disease of the central nervous system. Its aetiology remains unclear, and the most widely accepted theory of MS pathogenesis assigns the main role in the destruction of myelin to inflammation related to autoimmune reactions [42]. New keys to understanding MS pathogenesis have appeared after cloning the IgG repertoire directly from active plaques and periplaque areas of MS brain and from B-cells recovered from the cerebrospinal fluids of patients with MS with subacute disease [43]. It was found that anti-DNA Ab is a major component of the intrathecal IgG response of the MS patients and can promote important neuropathologic reactions in chronic disorders, such as MS and SLE [43].

Detection of a neurotropic effect in SCZ associated with damage of cellular membranes was explained by formation of brain autoantigens and auto-Abs [21,22]. Therefore, we could not exclude that, in the case of SCZ, the formation of antibodies to DNA may occur.

In this report, we use several methods to provide the first evidence of DNase activity of polyclonal IgGs isolated from the sera of patients with SCZ. In addition, we have analysed possible correlations of anti-DNA antibodies and relative activity of abzymes with DNase activity with various clinical forms of SCZ.

## 2. Material and methods

### 2.1. Chemicals, donors and patients

Most chemicals and proteins were from Sigma, and the Superdex 200 HR 10/30 column was from GE Healthcare. Sera of 20 patients (20–61 years old; average value 33.0 ± 7.4; 12 men and 8 women) with clinically definite SCZ (the total group includes 10 patients with positive and 10 patients with negative symptoms of the disease) were used to study DNase abzymes. Patients with positive symptoms were characterized by delusions, disordered thoughts and speech, and tactile, auditory, visual, olfactory and gustatory hallucinations, typically regarded as manifestations of psychosis [44]. Hallucinations were also typically related to the content of the delusional theme [45]. Negative symptoms are deficits of normal emotional responses or of other thought processes, and are less responsive to medication [46]. They commonly include flat expressions or little emotion, poverty of speech, inability to experience pleasure, lack of desire to form relationships and lack of motivation. Negative symptoms appear to contribute more to poor quality of life, functional ability and the burden on others than positive symptoms do [47]. The diagnosis was confirmed and its reliability was checked according to the standard international psychometric criteria PANSS (the Positive and Negative Syndrome Scale) for patients with SCZ including evaluation of positive, negative, general psychopathology parameters, as well as AIMS (Abnormal Involuntary Movement Scale) and CGI (Clinical Global Impression). Clinically verified diagnoses according to ICD-10 (International Classification of Diseases, 10th revision) were supplied to us by the doctors of the Department of Endogenous Disorders, Mental Health Research Institute (Tomsk, Russia). It was shown by specialists that the 20 patients with SCZ have had a negative history of typical systemic autoimmune rheumatic diseases.

For comparison, we used the sera of 20 healthy donors having negative history of autoimmune, rheumatologic, respiratory, cardiovascular, gastrointestinal, reproductive or nervous system pathology. Sera of 20 patients (27–60 years old; men and women) with clinically definite SLE described earlier [48,49] were also used for a comparison. The SLE diagnosis was confirmed and its reliability was checked according to the criteria developed by the American Rheumatoid Association [50,51].

### 2.2. IgG purification

Electrophoretically and immunologically homogeneous IgGs were obtained by sequential affinity chromatography of the serum proteins on protein G-Sepharose and FPLC gel filtration similar to [52–55]. The blood serum was loaded onto a protein G-Sepharose column (1 ml) equilibrated in buffer A (150 mM NaCl, 50 mM Tris–HCl, pH 7.5). The column was washed by buffer A to zero optical density (A_280_). Proteins bound non-specifically were eluted with the same buffer (3 ml) but containing 1% Triton X-100 and 0.5 M NaCl, and the column was washed with buffer A to zero optical density. IgGs were eluted with 0.1 M glycine–HCl (pH 2.6), the column fractions were collected into cooled tubes containing 25 µl of 1 M Tris–HCl (pH 8.8) and, finally, each fraction was additionally neutralized with this buffer and dialysed against 50 mM Tris–HCl (pH 7.5). The protein corresponding to the central part of the IgG peak was concentrated in the dialysis bag by air flow at 4°C and used in further purification. To protect the Abs from bacterial contamination they were sterilized by filtration through 0.2 µm Millex filter. Aliquots of the preparations for experiments conducted within one to two weeks after Abs purification were stored at 4°C, while IgGs using later than two weeks were frozen at −70°C.

IgGs were incubated in 50 mM glycine–HCl (pH 2.6) for 10 min at 25°C. Separation of the IgGs under ‘acid shock’ conditions was done by FPLC gel filtration on a Superdex 200 HR 10/30 column equilibrated with 10 mM glycine–HCl (pH 2.6) containing 0.1 M NaCl as described previously [52–55]. Fractions were collected, neutralized and sterilized as described above. After two weeks of storage at 4°C for refolding after the acid shock, the Abs were used in the activity assays as described below.

### 2.3. ELISA of anti-DNA Abs

The levels of anti-DNA Abs were determined using standard ELISA: plates with immobilized double-stranded (ds) and single-stranded (ss) DNA, horseradish peroxidase-conjugated mouse Abs against human IgG by test system ORGENTEC Diagnostika (Germany) [54]. It is known that Abs interacting with immobilized DNA can occur not only directly to DNA but also to DNA-binding enzymes as well as to any polyspecific immunoglobulins, but the ELISA approach, widely used in immunology, does not allow one to distinguish between such antibodies. Since the concentrations of Abs interacting with DNA in serum of different healthy donors and SCZ patients can vary greatly, first of all we analysed the dependence of final absorbance at 450 nm for several samples at dilutions of 2000-, 1000-, 500- and 100-fold in order to find operating range. Finally, 1000-fold dilution of the sera recommended by the manufacturer was found to be the optimal, and the relative levels of Abs interacting with single- and double-stranded DNA were estimated according to standard manufacturer's protocol. The coefficients of variation for the ELISA according to the producer's data vary from 5 to 15% depending on the concentration of Abs interacting with DNA.

The reaction was stopped with sulfuric acid, and optical density (A_450_) of the solutions was determined using an Epoch spectrophotometer (BioTek, USA). The relative concentrations of Abs interacting with DNA in the samples were expressed as a difference in the relative absorbance at 450 nm (average of three measurements) between the experimental and the control samples; controls using DNA without Abs and with IgGs from healthy humans not interacting with DNA (specific fraction of human Abs not interacting with DNA-cellulose) produced the same results. Finally, the concentration of IgGs interacting with DNA was calculated and the results were expressed in A_450_ ml^−1^ units.

### 2.4. DNase activity assay

DNA-hydrolysing activity was analysed using supercoiled (sc)DNA, as described earlier for analysis of DNase I, DNase II [56,57] and human serum catalytic antibodies [54,58,59]. The reaction mixture (20 µl) contained 18 µg ml^−1^ (6.1 nM; molecular mass of DNA 2.95 × 10^6^ Da) supercoiled DNA pBluescript, 5 mM MgCl_2_, 1 mM EDTA, 20 mM Tris–HCl (pH 7.5) and 0.005–0.2 mg ml^−1^ Abs (corresponding to the central part of the peaks after gel filtration), and was incubated for 0.5–10 h (standard time, 2 h) at 37°C. The cleavage products were analysed by electrophoresis in 1% agarose gel. The images of ethidium bromide-stained gels were captured on a Sony DSC-F717 camera and a relative amount of DNA in different bands was analysed using ImageQuant v. 5.2 (Molecular Dynamics). All quantitative measurements of the relative activity of Abs were performed according to the general methods of determination of enzyme-specific activities [60]. The activities of IgG preparations were determined as a decrease in the percentage of ssDNA converted from the initial supercoiled form to the relaxed form (and sometimes additionally to linear form), corrected for the distribution of DNA between these bands in the control (incubation of pBluescript in the absence of Abs). All measurements (initial rates) were taken within the linear regions of the time courses (15–40% of DNA hydrolysis) and a complete transition of the supercoiled plasmid to the nicked form was taken for 100% activity. If the activity was low (less than 5–10% of scDNA disappearance), the incubation was prolonged to 2–10 h, depending on the sample. If the degradation of supercoiled DNA after 1–2 h of incubation exceeded 50%, the concentration of Abs was lowered 2- to 100-fold, depending on the sample. This approach allowed normalization of the relative activity, as in the case of determination of the specific activity of enzymes [60], to any standard condition. The measured relative activities (RAs) for IgGs were normalized to standard conditions (0.1 mg ml^−1^ IgGs, 1 h).

### 2.5. *In situ* DNase activity assay

SDS-PAGE analysis of Abs (central part of the IgG peak after gel filtration; mixture of only 15 scz-IgGs containing no 17 000–17 400 Da light chains) for homogeneity under non-reducing conditions was performed in a 5–16% gradient gel containing 0.1% SDS, and for the polypeptide spectrum, in a reducing 12% gel containing 0.1% SDS and 50 mM dithiothreitol (Laemmli system) [54,59]. The polypeptides were visualized by silver staining and by Western blotting on a nitrocellulose membrane [54,59].

The DNase activity of IgG after SDS-PAGE was analysed in a gel containing calf thymus DNA (5 µg ml^−1^) under reducing and non-reducing conditions as in previous studies [54,59]. Before the electrophoresis, the IgG samples were incubated at 22°C for 10–20 min in 20 mM Tris–HCl (pH 7.5) containing 0.1% SDS. To restore the enzymatic activity after SDS-PAGE, SDS was removed by incubating the gel for 1 h at 22°C in 20 mM Tris–HCl (pH 7.5) and washing the gel five times with the same buffer. To refold the protein after SDS treatment and to assay for DNase activity, longitudinal slices of the gel were incubated at 25°C for 15–48 h in the reaction buffer containing 20 mM Tris–HCl (pH 7.5), 4 mM MgCl_2_ and 0.2 mM CaCl_2_. To visualize the products of DNA hydrolysis, the gel was stained with ethidium bromide. The same ethidium bromide-stained or parallel longitudinal slices were used to detect the position of IgG in the gel by Coomassie Blue staining.

### 2.6. MALDI-TOF mass spectrometry analysis of IgGs

IgGs were analysed by MALDI-TOF mass spectrometry (positive mode) using a Reflex III system (Bruker, Germany) equipped with a 337 nm nitrogen laser (VSL-337 ND, Laser Science, Newton, MA, USA), 3 ns pulse duration. Saturated solution of sinapinic acid in a mixture of 0.1% acetonitrile and trifluoroacetic acid (1 : 2) was used as matrix. For the analysis of antibodies, we have used the native IgGs (all fractions of IgG peak after gel filtration; all fractions demonstrated positive response with mouse Abs against human IgGs) and the same preparations after treatment with DTT (0.01 M) for 10 min at 90°C. To 1 µl of the reaction mixture containing the intact or treated IgGs 1 µl of solution containing matrix was added; the final mixture was spotted on the MALDI AnchorChip plate, air-dried and used for the analysis. Calibration of the MALDI mass spectrometry spectra was performed using the protein standards I and II (Bruker Daltonic, Germany) in the external and internal calibration mode.

### 2.7. Estimation of the kinetic parameters

The reaction mixtures contained the standard components and 4–200 nM supercoiled pBluescript DNA. The relative amount of DNA (%) in the bands corresponding to supercoiled (substrate) and relaxed DNA (product of the reaction) was estimated as described above and then the relative amount of relaxed DNA (nM) was calculated taking into account the concentration of DNA in every reaction mixture. The apparent *K*_m_ and *V*_max_ (*k*_cat_) values were calculated from the dependencies of *V* versus [DNA] by least-squares nonlinear fitting using Microcal Origin v. 5.0 software and presented as linear transformations using a Lineweaver–Burk plot [60]. Errors in the values were within 7–15%. The results are reported as mean ± standard deviation of at least three independent experiments for each sample of IgGs.

### 2.8. Statistical analysis

The results are reported as mean ± standard deviation of three independent experiments for each sample of IgGs. To check normality of the distribution law the criterion of Shapiro–Wilk's *W* test was used. Most of the sample sets did not meet the normal Gaussian distribution. Therefore, the differences between IgG samples of different groups were estimated using the Mann–Whitney test, and *p* < 0.05 was considered statistically significant. The median (M) and interquartile ranges (IQR) were estimated.

## 3. Results

### 3.1. Characteristic of patients

In this study, we have analysed the relative levels of Abs interacting with ssDNA and dsDNA and Abs DNase activity in the case of 20 patients with SCZ compared with 20 Abs from healthy donors. Some general characteristics of SCZ patients are given in table 1 and the Material and methods section.

**Table 1.** General characteristics of 20 patients with SCZ.

patient number	symptoms of disease	sex	age	duration of disease (years)
1	positive	male	54	19
2	positive	male	34	17
3	positive	male	34	12
4	positive	male	21	3
5	positive	male	29	1
6	positive	male	31	5
7	positive	male	35	13
8	positive	female	30	5
9	positive	female	26	7
10	positive	female	37	1
average value	positive		33.1 ± 5.7	8.3 ± 5.6
11	negative	male	21	5
12	negative	male	45	9
13	negative	male	34	11
14	negative	male	20	13
15	negative	male	35	3
16	negative	female	23	7
17	negative	female	34	14
18	negative	female	26	7
19	negative	female	61	24
20	negative	female	20	1
average value	negative		32.9 ± 9.9	9.4 ± 4.9
average value	positive + negative		32.5 ± 7.8	8.9 ± 5.2

### 3.2. ELISA of Abs interacting with DNA

**Figure 1.**
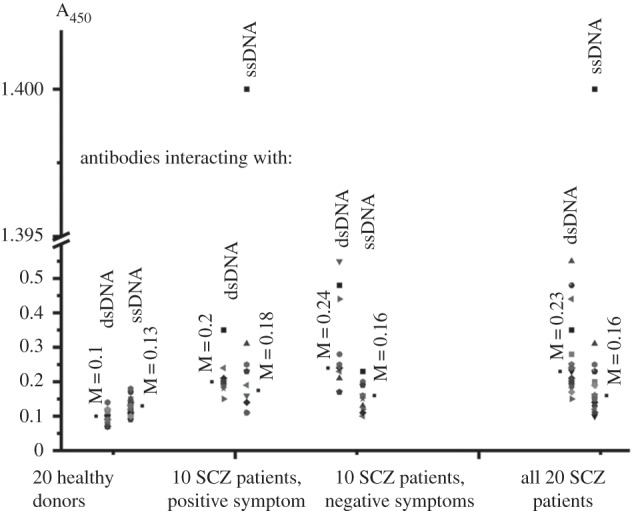
The distribution within different ranges of A_450_ values characterizing the levels of Abs interacting with ssDNA and dsDNA for healthy donors and SCZ patients with positive and negative symptoms. The points left and right of the given series of values correspond to the values of medians estimated using the Mann–Whitney test.

The generation of auto-Abs to self-antigens including DNA usually occurs not only in patients with autoimmune, viral and bacterial diseases but also in healthy humans [23,24,32–35,57]. We have estimated the relative levels of Abs interacting with DNA including the median (M) and interquartile ranges (IQR) (table 2). The distribution of A_450_ ml^−1^ values within possible ranges is shown in figure 1. The levels of Abs interacting with ssDNA (A_450_ ml^−1^) for 20 healthy donors were significantly detectable and varied from 0.07 to 0.14 specific units (mean ± s.d., average value −0.13 ± 0.02), and on average they were 1.3-fold higher (*p* = 6.3 × 10^−4^) than those for interacting with dsDNA varying from 0.1 to 0.18 (0.1 ± 0.02) (table 2). Similar values of A_450_ (0.1 ± 0.04 and 0.13 ± 0.09 A_450_ ml^−1^) for healthy donors were also obtained earlier [33,34]. The correlation coefficient (CC) between Abs interacting with scDNA and dsDNA in the case of healthy humans was 0.49 (table 2).

**Table 2.** The relative content of antibodies interacting with single- and double-stranded DNA in plasma of healthy donors and patients with SCZ.^a.^

	healthy donors			SCZ patients	
	levels of Abs interacting with dsDNA, A_450_ ml^−1^	levels of Abs interacting with ssDNA, A_450_ ml^−1^		levels of Abs to dsDNA, A_450_ ml^−1^	levels of Abs to ssDNA, A_450_ ml^−1^
number of healthy donors (sex)	1	2	number of SCZ patients (sex)	3	4
			positive symptoms		
1 (M)	0.07	0.12	1 (M)	0.35	1.4
2 (M)	0.1	0.11	2 (M)	0.19	0.11
3 (M)	0.12	0.11	3 (M)	0.2	0.31
4 (M)	0.1	0.11	4 (M)	0.19	0.16
5 (M)	0.1	0.13	5 (M)	0.24	0.19
6 (F)	0.09	0.11	6 (M)	0.15	0.11
7 (F)	0.07	0.11	7 (M)	0.21	0.14
8 (F)	0.1	0.09	8 (F)	0.2	0.23
9 (F)	0.08	0.13	9 (F)	0.34	0.25
10 (F)	0.12	0.17	10 (F)	0.18	0.11
average				0.23 ± 0.05	0.30 ± 0.22
M (IQR)^d^				0.20 (0.05)	0.18 (0.14)
cor. coeff.				groups 3–4 (0.71)	
			negative symptoms		
11 (M)	0.09	0.14	11 (M)	0.48	0.23
12 (M)	0.11	0.15	12 (M)	0.28	0.2
13 (M)	0.14	0.17	13 (M)	0.21	0.13
14 (M)	0.12	0.15	14 (M)	0.22	0.16
15 (M)	0.1	0.13	15 (M)	0.23	0.1
16 (F)	0.09	0.12	16 (F)	0.44	0.12
17 (F)	0.07	0.14	17 (F)	0.24	0.11
18 (F)	0.12	0.1	18 (F)	0.17	0.19
19 (F)	0.09	0.13	19 (F)	0.25	0.16
20 (F)	0.14	0.18	20 (F)	0.24	0.15
average	—	—		0.28 ± 0.07	0.16 ± 0.03
M (IQR)				0.24 (0.06)	0.16 (0.07)
cor. coeff.				groups 3–4 (0.3)	
			complete group of 20 patients		
average value	0.10 ± 0.02^b^	0.13 ± 0.02	average value	0.25 ± 0.07	0.23 ± 0.13
M (IQR)	0.10 (0.03)	0.13 (0.04)		0.23 (0.07)	0.16 (0.1)
cor. coeff.	total groups 1–2 (0.49)			total groups 3–4 (0.3)	
difference, *P*^c^	total groups 1 and 3 (1.0 × 10^−6^)		total groups 2 and 4 (0.05)		
	total group 1—pos. 3 (1.0 × 10^−5)^		total groups 2 and pos 4 (0.1)		
	total group 1—neg. 3 (1.1 × 10^−5)^		total group 2—neg. 4 (0.12)		
	pos 3 − pos 4 (0.15)		neg. 3 − neg. 4 (8.6 × 10^−4^)		
			total groups 3 and 4 (6.9 × 10^−4^)		

^a^For each value, a mean of three measurements is reported; the error of the determination of values did not exceed 7–10%.

^b^Average values are reported as mean ± s.e.

^c^Coefficient *P* was calculated using the Mann–Whitney test, *p* < 0.05 was considered statistically significant.

^d^The median (M) and interquartile ranges (IQR) were calculated using the Mann–Whitney test.

For the total group of 20 individual SCZ patients, the levels of Abs interacting with DNA varied in broad ranges (table 2, figure 1). The average level of Abs (A_450_ ml^−1^) interacting with ssDNA (range from 0.1 to 1.4; 0.23 ± 0.13) was only 1.1-fold lower (*p* = 6.9 × 10^−4^) than that for interacting with dsDNA (range from 0.15 to 0.44; 0.25 ± 0.07). The CC between the Abs interacting with ssDNA and dsDNA in the case of all SCZ patients was 0.3 (table 2). The average level of Abs interacting with dsDNA in the case of healthy donors is 2.5-fold lower (*p* = 1.0 × 10^−6^) than that for SCZ patients, while for Abs interacting with ssDNA it is lower only by a factor of 1.8 (*p* = 0.05) (table 2).

The distribution of A_450_ ml^−1^ values for ACZ patients with positive and negative symptoms is shown in figure 1. The relative average level of Abs interacting with dsDNA (0.23 ± 0.05 A_450_ ml^−1^) for patients with positive symptoms is 1.2-fold lower than that for patients with negative symptoms (0.28 ± 0.07 A_450_ ml^−1^) (table 2). At the same time, the average level of Abs interacting with ssDNA (0.3 ± 0.22 A_450_ ml^−1^) for patients with positive symptoms was higher than that for patients with negative symptoms (0.16 ± 0.07 A_450_ ml^−1^) 1.9-fold (table 2). Several SCZ patients with positive and negative symptoms are characterized by very high levels of Abs interacting with dsDNA and ssDNA (0.31–1.4 A_450_ ml^−1^), which are characteristic of 25–37% of patients with SLE (0.66 ± 0.48 and 0.51 ± 0.57 A_450_ ml^−1^, respectively) and with MS (0.39 ± 0.26 and 0.22 ± 0.18 A_450_ ml^−1^, respectively) [32,33]. Therefore, one cannot exclude that anti-DNA Abs can play an important role in pathogenesis not only for patients with SLE and MS (see above) but also in the case of some individuals with SCZ.

### 3.3. Purification and characterizing of IgGs

**Figure 2.**
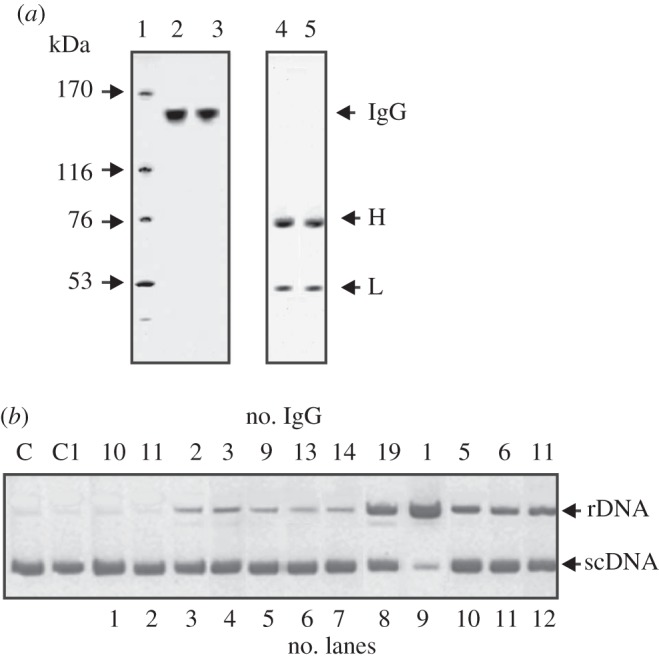
(*a*) SDS-PAGE analysis of IgG_mix_ (7 µg) corresponding to the mixtures of sch-IgG_mix_ (line 2) from the sera of 20 SCZ patients and healthy-IgG_mix_ (lane 3) corresponding to 20 healthy donors in a non-reducing 3–16% gradient gel or a reducing 12% gel (lanes 4 and 5, respectively) followed by silver staining. The arrows (lane 1) indicate the positions of MM markers. (*b*) Activity of IgGs from 12 SCZ patients (lanes 1–12) in the cleavage of double-stranded supercoiled (sc) pBluescript plasmid DNA leading to a formation of relaxed plasmid (rDNA). scDNA was incubated for 1 h at 37°C with electrophoretically homogeneous IgGs from nine patients (0.1 mg ml^−1^; lanes 3–8), IgGs from four other donors (0.03 mg ml^−1^; lanes 9–12), IgGs from two patients (0.2 mg ml^−1^ for 4 h; lanes 1 and 2), IgGs corresponding to the mixture of Abs from healthy donors (IgG_mix;_ 0.2 mg ml^−1^ for 4 h; lane C1). Lane C corresponds to scDNA incubated for 4 h without Abs.

In this work, electrophoretically and immunologically homogeneous IgG was purified from the sera of 20 healthy donors and 20 SCZ patients by sequential chromatography of the serum proteins on Protein A Sepharose under conditions that remove non-specifically bound proteins, followed by FPLC gel filtration in an acidic buffer destroying immune complexes as in previous studies [52–55]. To analyse an ‘average’ situation concerning homogeneity of IgGs, we have prepared a mixture of equal amounts of polyclonal IgGs (corresponding to central parts of the peaks after gel filtration, scz-IgG_mix_) from the sera of 20 SCZ patients and 20 healthy donors (healthy-IgG_mix_)**.** The homogeneity of the typical 150 kDa IgG_mix_ was confirmed by SDS-PAGE with silver staining, which showed a single band under non-reducing conditions and two bands corresponding to the H and L chains after reduction (figure 2*a*). The homogeneity of the typical control 150 kDa IgG_mix_ from the sera of 20 patients with SLE was shown earlier by SDS-PAGE with silver staining [48,49].

We first used mixture of 20 IgG preparations from healthy donors and confirmed the previously published data ([31–34,48,49] and references therein) that IgGs from healthy humans do not possess detectable DNase activity (figure 2*b*, lanes 10 and 11). Although the sera from the healthy donors contained auto-Abs interacting with DNA, they were inactive even after a 5 h incubation of scDNA in the presence of 0.2 mg ml^−1^ IgGs.

### 3.4. Analysis of IgGs by MALDI mass spectrometry

**Figure 3.**
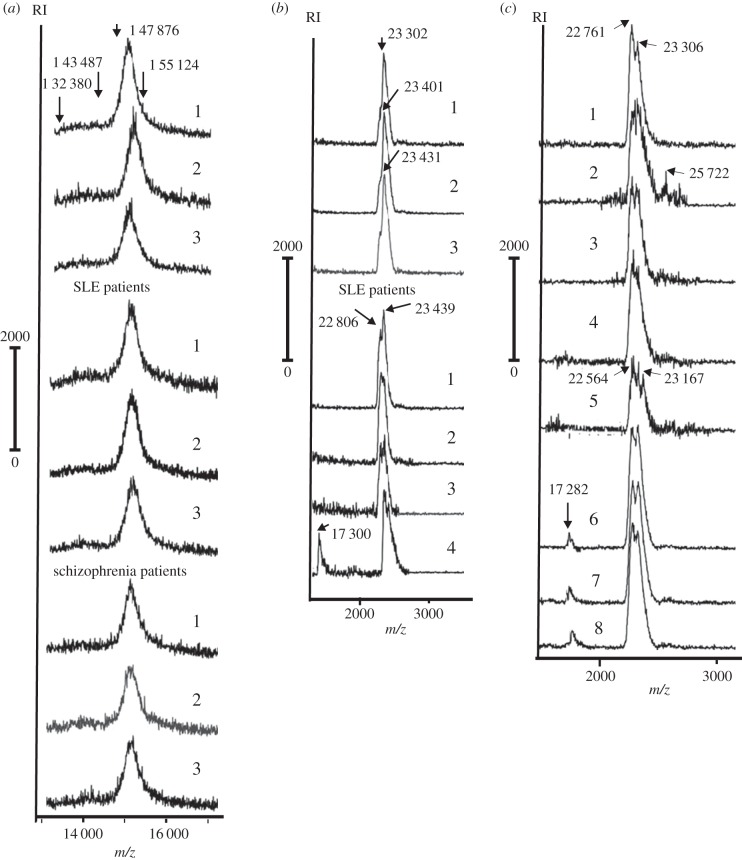
MALDI mass spectrometry spectra of (*a*) several intact IgGs, and (*b*,*c*) antibodies after complete reduction with DTT. IgGs from the sera of healthy donors (*a*,*b*), SLE (*a*,*b*) and SCZ patients (*a*,*c*) were used. See Material and methods section for other details.

It is known that determination of molecular masses (MMs) by SDS-PAGE can give only approximate values of them and proteins with comparable MMs form relatively wide protein bands including polyclonal IgGs of different protein sequences and levels of glycosylation (e.g. figure 2*a*). For more precise evaluation of the MMs of IgGs, we have used MALDI-TOF mass spectrometry. Figure 3*a* demonstrates three typical examples of MALDI mass spectrometry spectra of intact IgGs (H_2_L_2_) corresponding to healthy donors, SLE and SCZ patients. It should be mentioned that spectra of intact IgGs for healthy donors, SLE and SCZ patients are very similar and they lack any individual character. All spectra contain many peaks of singly charged IgG^+^ molecules (glycosylated in different degree) with MMs approximately from 143 487 to 155 124 Da, forming a single generalized peak with maximum at about from 147 877 to 149 537 Da. In addition, in all the spectra there are some relatively weak peaks with MMs from 132 380 to 143 487 Da. Thus, based on the data of the MALDI spectra data of the intact IgGs, it is impossible to distinguish between healthy donors and autoimmune patients. Taking this into account, we have treated the IgGs from all healthy donors, SLE and SCZ patients with DTT. MALDI spectra of heavy chains (H^+^) usually contain two peaks of different intensity with somewhat comparable MMs, but (similarly to spectra of intact IgGs) they were uninformative for discrimination of IgGs of healthy donors and autoimmune patients. More informative and interesting were spectra of IgG light chains.

The spectra of the IgG light chains of all 20 healthy donors were relatively homogeneous and characterized by one peak with only one maximum; figure 3*b* demonstrates three spectra, which are typical for all 20 healthy donors. In contrast to healthy donors, all spectra of IgG light chains corresponding to autoimmune SLE patients (e.g. numbers 1–3) demonstrated a peak with two maxima of a comparable intensity. The new brightly pronounced peaks are characterized by lower MMs (22 806–23 000 Da) compared with the single peaks of healthy donors (23 302–23 431 Da) and the second peaks of IgG light chains of SLE patients (23 439–24 000 Da) (figure 3*b*). In addition, the MALDI spectra of the IgG light chains of five SLE patients contained an additional brightly pronounced peak at 17 000–17 400 Da (for example, sample number 4; figure 3*b*). Interestingly, MALDI spectra of light chains of IgGs of SCZ patients had a lot in common with those of SLE patients, but not with healthy donors. All MALDI spectra of light chains of 20 SCZ patients demonstrated at least two maxima, which are comparable in their intensity (figure 3*c*). In addition, four of 20 SCZ patients were characterized with three pronounced maxima (e.g. sample number 5). Moreover, similar to SLE antibodies, 4 of 20 SCZ IgGs contained light chains with MMs from 17 200 to 17 300 Da (e.g. figure 3*b*, numbers 6–8). The spectra of IgG light chains of several SCZ patients are additionally characterized by small peaks corresponding to MMs higher than 23 300 Da (e.g. 25 722 Da, samples 2–5). Taken together, it is obvious that IgG light chains of SCZ patients may differ significantly from those for healthy donors, but at the same time they are similar and even in some cases a little more complicated than those for SLE patients. One cannot exclude that 17 kDa light chains of IgGs may appear in the sera of some SLE and SCZ patients due to their hydrolysis in the result of post-translational processing. These data suggest that, similarly to SLE patients, SCZ patients are characterized by obvious autoimmune processes leading to formation of various IgGs, which are different from those for healthy donors.

### 3.5. Application of the strict criteria

**Figure 4.**
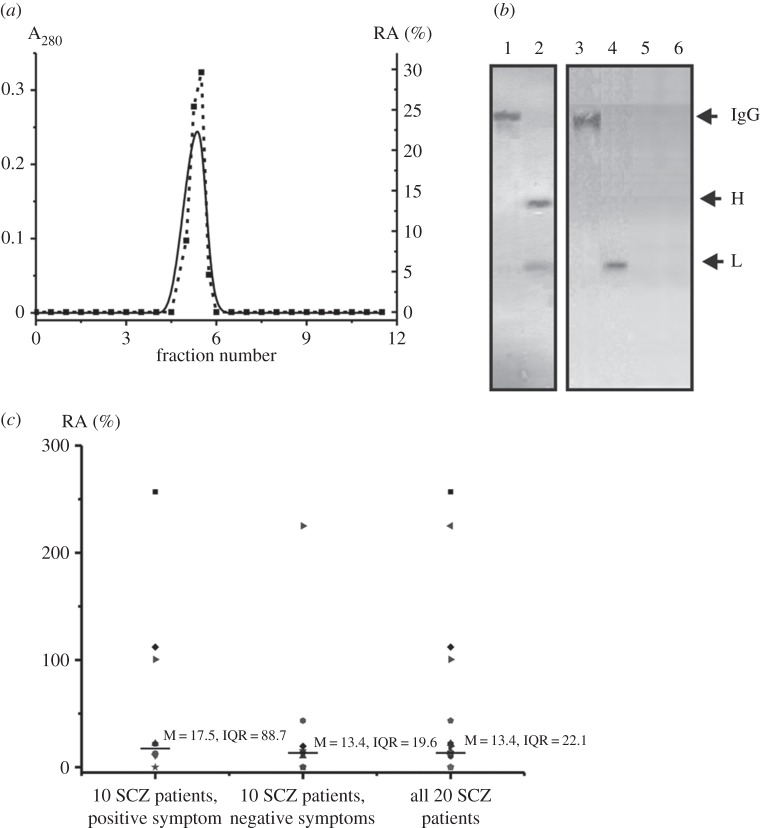
Application of the strict criteria to prove that the DNase activity of Abs is intrinsic property of SCZ IgG_mix_. (*a*) FPLC gel filtration of sacs-IgG_mix_ on a Superdex 200 column in an acidic buffer (pH 2.6) after Abs pre-incubation in the same buffer: solid line, absorbance at 280 nm (A_280_; square), relative activity (RA, %) of IgGs in the hydrolysis of scDNA. A complete hydrolysis of 18 µg ml^−1^ scDNA for 2 h was taken for 100%. The error in the initial rate determination from two experiments in each case did not exceed 7–10%. (*b*) Assay of DNase activity of scz-IgG_mix_ from SCZ patients in-gel containing thymus DNA before (lane 3) and after Abs reduction with DTT (lane 4); lane 5 corresponds to healthy-IgG_mix_ before treatment with DTT. DNase activity was revealed by ethidium bromide staining as a dark band on the fluorescent background. There was no revealed DNase activity of healthy-IgG_mix_ before reduction (lane 5). A part of the gel was stained with Coomassie R250 to show the position of intact scz-IgG_mix_ before (lane 1) and its free heavy and light chains after reduction (lane 2). (*c*) The distribution within different ranges of the RAs (in the hydrolysis of scDNA) corresponding to SCZ patients with positive and negative symptoms. Solid lines show the medians estimated using the Mann–Whitney test. For details, see Material and methods section.

To prove that the DNase activity of IgGs from the sera of SCZ patients belongs to the Abs and is not due to co-purifying enzymes, we have applied several previously developed strict criteria [31–34,61]. They may be summarized as follows: (i) the IgG_mix_ (corresponding to the central parts of the peaks after gel filtration) was electrophoretically homogeneous (figure 1*a*); (ii) gel filtration of IgGs under conditions dissociating strong non-covalent complexes in an acidic buffer (pH 2.6, figure 4*a*) did not eliminate the DNase activity, and the peaks of the activity tracked exactly with the intact IgGs; and (iii) immobilized mouse polyclonal IgGs against the light chains of human IgGs completely absorbed the DNase activity.

Among these criteria there is one which, if it is carried out, all other criteria are also carried out. To exclude possible artefacts due to hypothetical traces of contaminating enzymes, an scz-IgG_mix_ preparation (mixture of only 15 scz-IgGs containing no 17 000–17 400 Da light chains) was subjected to SDS-PAGE in a gel co-polymerized with calf thymus DNA, and their DNase activity was detected by incubating the gel in the standard reaction buffer (figure 4*b*). Ethidium bromide staining of the gels after the electrophoresis and refolding of IgGs revealed sharp dark bands against a fluorescent background of DNA only in the position of intact IgG before (lane 3) and only in the position of light chains after its reduction with DTT (lane 4). There was no detected DNase activity of healthy-IgG_mix_ before (lane 5) and after Abs reduction with DTT (lane 6).

Canonical human DNases have MMs (35–36 kDa) significantly lower than the intact IgGs (150 kDa), but higher than free light chains of IgGs (22–25 kDa). Since SDS dissociates all protein complexes, the detection of the activity in the gel zones corresponding only to intact IgGs and its light chains, together with the absence of any other activity band or protein band (figure 4*b*), provides direct evidence that SCZ IgGs hydrolyse DNA and are not contaminated by canonical DNases. Several other strict criteria were also fulfilled (see below).

### 3.6. Estimation of the relative DNase activity

We have confirmed that the DNase activity is an intrinsic property of IgGs from SCZ patients (see above) and that the Abs obtained by chromatography on Protein G-Sepharose followed by FPLC gel filtration can be used to evaluate their relative activity without additional purification. To estimate the DNase activity quantitatively, we have found the concentration for each IgG preparation (see Material and methods section) for conversion of scDNA into the relaxed form without fragmentation after 0.5–10 h of incubation (e.g. lanes 3–7 of figure 2*b*). The relative activities of IgGs from the sera of SCZ patients significantly varied from patient to patient, but 16 of 20 samples (80%) had detectable or high DNase activity. The measured relative activities (RAs, %) for IgGs were normalized to standard conditions (0.1 mg ml^−1^ IgGs, 1 h) and a complete transition of scDNA to its relaxed form was taken for 100% DNase activity (table 3). The distribution of the RAs for IgGs of different SCZ patients is shown in figure 4*c*.

**Table 3.** The relative efficiency (%) and the apparent *k*_cat_ values characterizing hydrolysis of scDNA by IgGs from the sera of SCZ patients.^a^

number of patients (sex)	Abs to dsDNA, A_450_ ml^−1^	Abs to ssDNA, A_450_ ml^−1^	relative hydrolysis of DNA (%)	*k*_cat_ × 10^5^min^−1^
	1 (3 in table 2)	2 (4 in table 2)	3	4
	positive symptoms (PS)			
1 (M)	0.35	1.4	257^d^	39.6^c^
2 (M)	0.19	0.11	11.7	1.8
3 (M)	0.2	0.31	22.2	3.4
4 (M)	0.19	0.16	11	1.7
5 (M)	0.24	0.19	12	1.9
6 (M)	0.15	0.11	100.4	15.5
7 (M)	0.21	0.14	112	17.3
8 (F)	0.2	0.23	22	3.4
9 (F)	0.34	0.25	13	2.0
10 (F)	0.18	0.11	0	0
average (PS)^b^	0.23 ± 0.05	0.30 ± 0.22	56.1 ± 60.2	8.7 ± 9.3
M (IQR) (PS)^e^	0.20 (0.05)	0.18 (0.14)	17.5 (88.7)	2.7 (13.7)
correl. coeff. (PS)	groups 1–2 (0.71)		1–3 (0.46)	2–3 (0.84)
	negative symptoms (NS)			
11 (M)	0.48	0.23	0^d^	0
12 (M)	0.28	0.2	0	0
13 (M)	0.21	0.13	10.4	1.6
14 (M)	0.22	0.16	15.0	2.3
15 (M)	0.23	0.1	13.3	2.1
16 (F)	0.44	0.12	225	34.7
17 (F)	0.24	0.11	19.6	3.0
18 (F)	0.17	0.19	13.6	2.1
19 (F)	0.25	0.16	43.4	6.7
20 (F)	0.24	0.15	0	0
average (NS)	0.28 ± 0.07	0.16 ± 0.03	34.0 ± 40.1	5.3 ± 6.2
M (IQR) (NS)	0.24 (0.06)	0.16 (0.07)	13.4 (19.6)	2.1 (3.0)
average, total group	0.25 ± 0.07	0.23 ± 0.13	45.1 ± 50.4	7.0 (7.9)
M (IQR), total group	0.23 (0.07)	0.16 ± 0.1	13.4 (22.1)	2.1 (3.4)
corr. coeff. (NS)	1–2 (0.3)	1–3 (0.5)	2–3 (0.35)	
corr. coeff.	1–2 (0.3)		2–3 (0.62)	
complete group		1–3 (0.4)		

^a^For each value, a mean of three measurements is reported; the error of the determination of values did not exceed 7–10%.

^b^Average values are reported as mean ± s.e.; they were recalculated to standard conditions and complete hydrolysis of 18 µg ml^−1^ scDNA after 1 h of incubation in the presence of 0.1 mg ml^−1^ IgG was taken for 100%.

^c^The average apparent *k*_cat_ values of the reaction of the hydrolysis of DNA at its fixed not saturated concentration (18 µg ml^−1^ or 6.1 nM) were calculated using average RA values: *k*_cat_ *=* *V* (M min^−1^)/[IgGs] (M).

^d^Statistical significance of differences in DNase activity between schizophrenia patients with positive and negative symptoms (*p* = 0.026).

^e^The median (M) and interquartile ranges (IQR) were calculated using the Mann–Whitney test.

Finally, to compare average values for Abs corresponding to different groups of SCZ patients with DNase IgGs from patients with other diseases we calculated the apparent *k*_cat_ values of the reaction at the fixed concentration of DNA for every IgG preparation, *k*_cat_ *=* *V* (M min^−1^)/[IgGs] (M) (table 3).

### 3.7. Determination of the kinetic parameters

**Figure 5.**
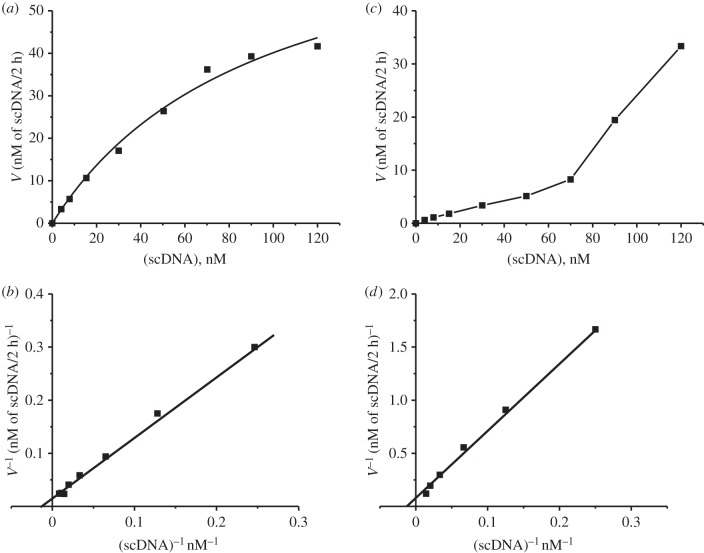
Dependencies of relative rates of the substrate hydrolysis on the concentration of scDNA and determination of the *K*_m_ and *V*_max_ values for scDNA using the Lineweaver–Burk plot in the case of (*a*,*b*) IgG19 and (*c*,*d*) IgG6, which were used in concentrations 240 and 33 nM, respectively. Panel (*b*) corresponds to the data of (*a*) in reverse coordinates. Panel (*d*) shows to the first part of *V* dependency on [scDNA] of (*c*) in reverse coordinates. Reactions were performed as described in the Materials and methods section. The error in the initial rate determination from two experiments at each substrate concentration did not exceed 7–12%.

One of the criteria for classifying the activity directly to antibodies is their higher affinity for DNA as compared with canonical DNases [31–34,40]. Therefore, we have estimated the *K*_m_ and *V*_max_ (*k*_cat_) values for scDNA hydrolysis using three preparations of SCZ IgGs. The first IgG preparation (number 19) corresponds to patients with negative symptoms of SCZ, while the second and third (numbers 1 and 6) corresponds to SCZ patients with positive symptoms. The dependency of the initial rate on the plasmid DNA concentration in the reaction catalysed by IgG1 and IgG19 is consistent with Michaelis–Menten kinetics (e.g. figure 5*a*,*b*). The *K*_m_ and *k*_cat_ values were determined in the case of IgG19: *K*_m_ = 95 ± 18 nM, *k*_cat_ = (2.7 ± 0.3) × 10^−3^ min^−1^. IgG1 demonstrated nearly the same *K*_m_ = 85.0 ± 12.0 nM, but higher *k*_cat_ = (7.9 ± 0.5) × 10^−3^ min^−1^*.* The IgG6 demonstrated (under the same conditions) more complicated dependencies, corresponding to a sum of two hyperbolic curves of abzyme saturation with DNA substrate (e.g. figure 5*c*,*d*). The first hyperbolic curve corresponds to *K*_m_ = 80.0 ± 12.0 nM and *k*_cat_ = (3.0 ± 0.3) × 10^−3^ min^−1^, while it was difficult to determine these values in the case of the second part of the dependency corresponding to lower *K*_m_ and higher *k*_cat_ values (figure 5*d*).

## 4. Discussion

We have analysed for the first time the levels of Abs interacting with DNA and DNase activity of IgGs in the plasma of patients with SCZ and compared them with those for healthy donors. Interestingly, for healthy donors the CC between the levels of Abs interacting with dsDNA and ssDNA is equal to 0.49 (table 2). At transition from healthy donors to patients with positive symptoms of SCZ the CC (0.71) between the levels of Abs interacting with dsDNA and ssDNA is increased by a factor of 1.4 (*p* = 1.0 × 10^−5^) and becomes 2.4-fold higher than that for patients with negative symptoms (CC = 0.3) (*p* = 1.1 × 10^−5^) (table 2). Overall, for 20 patients with SCZ, the CC between dsDNA (0.62) and RAs is 1.6-fold higher than that for ssDNA (0.4) and RAs (table 3). It should be mentioned that in the case of several previously analysed autoimmune and viral pathologies the CC between Abs interacting with dsDNA and their relative DNase activity varied in the range 0.4–0.8 [31–34].

MALDI mass spectrometry analysis of light chains of SCZ patients demonstrated that their spectra were similar to those for autoimmune SLE patients and were significantly different from the spectra of IgG light chains of healthy donors (figure 3).

It was shown that, as in the case of different autoimmune patients [31–34,40], mouse IgGs with DNase, proteolytic and amylase activities [58] are the earliest statistically significant markers of autoimmune pathology, and these activities are detectable even at the stage of pre-disease, when there are no visible markers of SLE or other pathologies and changes in proteinuria, and the anti-antigen titres including DNA are within the typical ranges of these indicators for healthy mice. A similar result was observed for SLE and MS patients [31–34]. Therefore, a detectable level of abzyme activities can be considered as an indicator even of the pre-disease (beginning of the pathology) and obvious pathology conditions of spontaneous AIDs [31–34,58]. As mentioned above, SCZ is not attributed to the typical AIDs [21,22].

We applied several previously developed strict criteria [61] (figure 4). The data reported in this paper provide strong evidence that DNase activity is an intrinsic property of IgGs presenting in the sera of SCZ patients: it is not due to copurifying enzymes. It was shown that increased concentration of Abs interacting with dsDNA is higher than that for healthy donors in the case of 36% of SLE patients, but 90–95% of Abs effectively hydrolyse DNA [31–34,40]; DNase abzymes were found in approximately 85–90% of MS patients [34]. Maximal values of A_450_ corresponding to Abs interacting with ssDNA and dsDNA of the group of 20 healthy donors used by us are relatively low (0.14 and 0.18 A_450_ ml^−1^, respectively) (table 2). However, it can be assumed that these values for individual healthy donors can sometimes reach values as high as 0.2 and 0.23 A_450_ ml^−1^ [31–34]. Taking this into account, 6 of 20 SCZ patients (approximately 30%) demonstrate a higher level of Abs interacting with dsDNA and ssDNA in comparison with healthy donors, which is comparable with that for SLE patients (36%) [23]. Abs with DNase activity was revealed in 80% of SCZ patients, which is also comparable with that for SLE and MS patients [31–34]. The data obtained may indicate that in some patients with SCZ autoimmune processes in varying degrees may occur.

As one can see from table 3, the relative average activity (percentage of the hydrolysis or *k*_cat_ values) for patients with positive symptoms (0–257%; average value 56.1 ± 60.2%) is about 1.6-fold higher (*p* < 0.05.) than that for patients with negative (0–225%; average value 34.0 ± 40.1%) symptoms. The RAs for individual patients vary in very wide ranges and do not correspond to normal (or Gaussian) distribution, and the medians are significantly different in comparison with the average RA values (table 3). The group of patients with positive SCZ symptoms contains 3 preparations with increased RA (100.4–257%) and one preparation with zero activity, while the group of patients with negative symptoms contains only one IgG with high activity, but three preparations without activity.

Interestingly, a clear correlation of the RAs with duration of SCZ is not observed (tables 1 and 2). Autoimmune and complex immunologic processes determined by genetic predisposition to immunoregulation disturbances are important in the pathogenesis of SLE [62]. In addition, for each MS patient, the ‘relative stability’ of different organs and their functions to the destructive effect of transient immune system errors can be significantly different depending on the genetic background and many environmental stress factors, including geographical ones [62,63]. On the one side, recent genome-wide association studies have shown that both common alleles of small effect and rare alleles of moderate to large effect contribute to the high genetic heritability of SCZ [64]. At the same time, it was shown that subtle abnormalities of cerebral anatomy (namely, small anterior hippocampi and enlarged lateral and third ventricles) are consistent with neuropathologic features of SCZ and that they (at least in part) are not genetic [65]. One cannot exclude that patients with a genetic predisposition to SCZ can exhibit greater susceptibility to the development of autoimmune processes, and therefore can demonstrate higher levels of Abs interacting with DNA and a higher activity of DNase abzymes. Furthermore, for individual SCZ patients, depending on different factors, the development of autoimmune reactions may have a very specific character. However, no definitive conclusions concerning the pathogenic role of DNA-binding antibodies in SCZ currently are possible because of antibody polyreactivity.

Usually, the active centre of abzymes with different activities is localized on the light chains of Abs [31–34]. In addition, isolated light chains of IgGs hydrolyse very different substrates including DNA. However, there is one example of abzymes (recombinant variable fragment of Abs of autoimmune-prone MRL-lpr/lpr mice) for which the DNase centre is located at the interface between the light and the heavy chains, and after separation both these chains are able to hydrolyse DNA [66]. Figure 4*b* demonstrates that DNase centres of SCZ IgGs (similarly to abzymes from the sera of patients with different AIDs) are located on the light chains, while heavy chains of Abs are inactive.

Different abzymes usually demonstrate significantly higher affinity to substrates in comparison with canonical DNases and proteases [31–34]. The affinity of the scDNA substrate for SCZ IgGs was (in terms of *K*_m_ values) in the range of 80–95 nM (figure 5), which corresponds to typical *K*_d_ (and *K*_m_) values for Ab–antigen interactions, and is about 3–4 orders of magnitude higher than affinity of scDNA for DNase I (*K*_m_ = 46–58 µM) [67]. These *K*_m_ values for scDNA to SCZ abzymes are comparable with the *K*_m_ for plasmid DNA (43 nM) reported previously for IgG from SLE patients [67].

It is interesting that IgG6 demonstrates complicated dependence of the relative rate on the concentration of DNA, consisting of a sum of at least two hyperbolic parts. At the same time, at the onset of AIDs the repertoire of abzymes is usually relatively small and abzymes can be characterized by only one pair of *K*_m_ and *k*_cat_ values. However, with the disease progression the number of abzymes with the same activity usually increases greatly, and the generation of catalytically diverse abzymes with very different activities and functions is often observed [31–34,40]. Sometimes polyclonal Abs demonstrate from two to seven different *K*_m_ and *k*_cat_ values. It was shown that different patients (and animals) may have a relatively small or an extremely large pool of polyclonal nuclease Abs containing different relative amounts of light chains of *κ*- and *λ*-types, demonstrating maximal activity at various optimal pHs, having a different net charge, activated or not by different metal ions, characterized by different substrate specificities and hydrolysis of substrates by IgGs of all four subclasses (IgG1–IgG4) [31–34,40]. It was recently shown that 34 of 78 recombinant monoclonal light chains (44%) corresponding to SLE patients have efficiently hydrolysed DNA demonstrating various pH optima and dependences on various metal ions [68,69]. Therefore, the detection of several abzymes with different properties should not be surprising since it is a typical phenomenon.

The catalysis mediated by artificial abzymes is usually characterized by the *k*_cat_ values being 10^2^- to 10^6^-fold lower than in the case of canonical enzymes ([31–34,40] and references therein). Importantly, the significantly higher affinity of substrates for abzymes compared with canonical enzymes with the same functions should lead to lower *k*_cat_ values, as the higher affinity prolongs the time of Ab–substrate complex existence and as a consequence decreases the catalyst turnover. The known *k*_cat_ values for natural abzymes from autoimmune patients vary in the range 10^−6^–40 min^−1^ [31–34,40,67].

Overall, the relative activities of DNase Abs from patients with different diseases increase approximately in the order: diabetes < viral hepatitis ≈ tick-borne encephalitis < polyarthritis ≤ Hashimoto's thyroiditis < AIDS ≤multiple sclerosis < SLE ([31–34,40] and references therein). The apparent *k*_cat_ values ((2.8–7.9) × 10^−3^ min^−1^) for three IgG preparations (figure 5) were 10^2^- to 10^3^-fold lower than the highest *k*_cat_ values for hydrolysis of scDNA by SLE IgGs with maximal activity [31–34,67], but they were comparable with the *k*_cat_ values for DNase IgGs from patients with tick-borne encephalitis [54] and viral hepatitis [59]. As the specific activities were calculated using the total concentration of purified IgGs, the specific DNase activities of the individual monoclonal subfractions in the IgGs pool may be higher than those of total polyclonal IgGs (table 2, figure 5).

In summary, for the first time, we have shown here that polyclonal IgGs from the sera of SCZ patients have high affinity for DNA and possess DNase activity. Abs against DNA are characteristic of SLE patients, but could at a first glance be considered non-specific side-products of the autoimmune processes in the sera of patients with many other autoimmune and viral diseases. We have shown previously that the appearance of abzymes hydrolysing DNA is among the earliest and clear signs of autoimmune reactions [31–34,40]. In addition, light chains of IgGs from SCZ patients are similar to those of SLE patients, but not to the chains of healthy donors. Therefore, one cannot exclude that abzymes with DNase activity may also be somewhat important for development of SCZ.
